# Synthesis and In Vitro Characterization of Fe^3+^-Doped Layered Double Hydroxide Nanorings as a Potential Imageable Drug Delivery System

**DOI:** 10.3390/ma10101140

**Published:** 2017-09-27

**Authors:** Lijun Wang, Yusen Wang, Xiaoxia Wang

**Affiliations:** School of Chemistry and Chemical Engineering, Shaoxing University, Shaoxing 312000, China; wys0_0@163.com (Y.W.); xxwang0119@163.com (X.W.)

**Keywords:** nanostructure, layered double hydroxides, MRI, drug carrier

## Abstract

Highly dispersed Fe^3+^-doped layered double hydroxide (LDH-Fe) nanorings were obtained by a simple coprecipitation-acid etching approach. The morphology, structure, magnetic resonance imaging (MRI) performance in vitro, drug loading and releasing, Fe^3+^ leakage, and cytotoxicity of the as-prepared LDH-Fe nanorings were characterized. The LDH-Fe nanorings showed good water dispersity and a well-crystallized structure. The DLS average size of nanoparticles was measured to be 94.5 nm. Moreover, the MRI tests showed a favourable T_1_-weighted MRI performance of the LDH-Fe nanoring with r_1_ values of 0.54 and 1.68, and low r_2_/r_1_ ratios of 10.1 and 6.3, pre- and after calcination, respectively. The nanoparticles also showed high model drug (ibuprofen) loading capacities, low Fe^3+^ leakage, and negligible cytotoxicity. All these results demonstrate the potential of LDH-Fe nanorings as an imageable drug delivery system.

## 1. Introduction

In personalized medicine, a real-time evaluation of the curative effect and timely adjustment of the treatment strategies are especially needed to realize the maximization of the efficacy and minimization of side effects [1,2]. Imageable drug delivery systems, which could monitor the drug transport in real time and assess the drug accumulation and even therapeutic effects at the target sites, have attracted much attention in the field of biomedicine in recent years [3,4,5,6,7,8,9,10,11].

Layered double hydroxides (LDHs), composed of stacked mixed divalent and trivalent metal hydroxide layers and interlaminar anions and bound water [12,13,14,15], have been widely studied as drug delivery carriers owing to their easy preparation, low toxicity, good biocompatibility, and high anionic drug loading capacity, etc. [14,16,17,18,19,20,21,22,23,24,25]. Furthermore, functional cations could be doped in the crystal structure of LDHs for bioimaging through a facile coprecipitation method, e.g., Gd^3+^ doped LDH could be used for MR imaging and Eu^3+^ doped LDH for optical imaging [23,26]. Therefore, LDHs can be used as a potential and promising functional carrier for the integration of both therapeutic and imaging agents through special structure designs.

Magnetic resonance imaging (MRI) is one of the most powerful non-invasive diagnostic technologies and can realize the high resolution imaging of soft-tissue [27,28]. In general, MRI contrast agents fall into two categories, T_1_-weighted and T_2_-weighted agents. Compared with T_2_-weighted MRI contrast agents, T_1_-weighted agents have a better imaging quality [29,30]. For example, the bright T_1_-weighted MR images are more effective in the diagnosis of normal and lesion tissues and have a better resolution than the T_2_-weighted ones [31,32,33]. In our previous work, paramagnetic Gd^3+^ with seven unpaired electrons as the T_1_-weighed MRI functional component was doped in the LDH crystal structure, and the obtained LDH-Gd showed an excellent T_1_-weighed imaging performance in vitro and in vivo, and could effectively monitor the biodistributions of the LDH drug carrier in mice and rats after a tail intravenous injection [26]. However, potential toxic Gd^3+^ leaky which could cause nephrogenic systemic fibrosis in patients [34,35,36] restricted the widespread application of LDH-Gd as an MRI contrast agent.

Fe and Mn are essential elements in biology, and paramagnetic Fe^3+^ and Mn^2+^ with five unpaired electrons as functional components in the nanoparticle carriers could also realize an excellent MRI performance [32,37,38]. However, Mn^2+^ doped in LDH in the form of Mn(OH)_2_ is not stable and is easily oxidized to high-valent species which do not possess the imaging function. Fe-based MRI contrast agents are usually used as effective T_2_-weighted contrast agents due to their extraordinarily high r_2_/r_1_ ratio [39]. In recent years, Fe-based T_1_-weighted contrast agents have been attempted by enhancing the T_1_-weighted MR imaging and meanwhile suppressing the T_2_-weighted MR imaging to obtain a better resolution in the diagnosis of disease [29,30,37,40,41,42]. Therefore, developing Fe^3+^ modified LDH drug carriers with favourable T_1_-weighted MR imaging characteristics and high biological safety is especially expected.

Herein, we report the synthesis of Fe^3+^ doped LDH nanorings (L-Fe-NR) through acid-etching of the pre-synthesized discoid Mg-Al(Fe)-CO_3_ LDH (L-Fe) by a one-step coprecipitation method and its favourable T_1_-weighted MRI performance and inherent drug carrier attributes. The doped Fe^3+^ is dispersed discretely in the LDH crystalline structure which leads to the volume magnetic anisotropy reduction and surface spin disorders of the nanoparticles, and thus could strongly reduce the T_2_-weighted imaging effect. The Fe^3+^ content on the surface of LDH can be substantially increased by acid etching due to the increased surface area of L-Fe-NR compared to L-Fe and more surface Mg^2+^ dissolved from LDH-Fe in the acid media than the surface Al^3+^ and Fe^3+^, which is favorable for the T_1_-weighted imaging. Meanwhile, the L-Fe-NR shows good water dispersibility and remains a well-crystallized layered structure after the acid etching, and thus anionic drug molecules could be encapsulated effectively in its interlayers. Furthermore, the L-Fe nanoring also shows negligible Fe^3+^ leakage and low cytotoxicity. This in vitro characterization demonstrates the potential of L-Fe-NR nanorings as a T_1_-weighted MRI contrast agent and a simultaneous drug carrier. Finally, a possible mechanism has been proposed for the LDH nanoring formation, which may provide a reference for the synthesis of LDH with different morphologies.

## 2. Experimental Section

### 2.1. Materials

Magnesium nitrate hexahydrate (≥99.0%), aluminum nitrate nonahydrate (≥99.0%), iron nitrate nonahydrate (≥98.5%), sodium nitrate (≥99.0%), nitric acid (65~68%), sodium hydroxide (≥96.0%), sodium bicarbonate (≥99.5%), and ethanol (≥99.7%) were all purchased from Sinopharm Chemical Reagent Co., Shanghai, China. All the chemical reagents were used directly without further purification. Ultra-pure deionized water from an ELGA system was used in all experiments.

### 2.2. Preparation of L-Fe

L-Fe was synthesized according to a doping method as reported previously [22,23,26]. In detail, 200 mL of mixed solution of Mg(NO_3_)_2_·6H_2_O (1.638 g), Al(NO_3_)_3_·9H_2_O (1.140 g), and Fe(NO_3_)_3_·9H_2_O (0.065 g) was titrated with a mixed alkaline solution of 2.0 g NaOH and 4.2 g NaHCO_3_ in 100 mL of deionized water at 25 °C until the final pH of the solution reached 9.5. After hydrothermal treatment at 150 °C for 24 h, the participate was concentrated by centrifugation, decanted, washed with deionized water three times, and vacuum-dried. To investigate the forming process of L-Fe, the intermediate was taken for TEM and EDS analysis. Before hydrothermal treatment at 150 °C, the solution pH was adjusted to 6.8 and 9.5, respectively. The products were collected by centrifugation as intermediate and donated as L-Fe-Pre-1 and L-Fe-Pre-2, respectively. After hydrothermal treatment at 150 °C for 1 h, the product was concentrated by centrifugation and donated as L-Fe-Pre-3, respectively.

### 2.3. Preparation of L-Fe-NR

A total of 100 mg of L-Fe was dispersed in 100 mL of aqueous solution containing 0.75 M NaNO_3_ and 0.0018 M HNO_3_ by ultrasonic treatment for 10 min, and then stirred for 12 h at ca. 25 °C. The obtained sample was recovered by centrifugation, decanted, washed with deionized water thrice, and then dispersed in 90 mL of deionized water. To investigate the property change of L-Fe in the acid etching process, 100 mg of L-Fe was treated with 100 mL of acid etching solution with HNO_3_ concentrations of 0.00045 M, 0.00090 M, and 0.00180 M, respectively. The obtained products were donated as L-Fe-AE-1, L-Fe-AE-2, and L-Fe-NR, respectively.

### 2.4. Characterization

Transmission electron microscopy (TEM) images were acquired using a JEM-2100F field-emission electron microscope (JEOL Ltd., Tokyo, Japan) operating at an accelerating voltage of 200 kV. An X-ray diffraction (XRD) pattern was obtained on a D/Max-2200 PC X-ray diffractometer (Rigaku Corporation, Tokyo, Japan) with Cu target irradiation operating at 40 kV and 40 mA. The UV-Vis absorbance spectra were recorded on a Shimadzu UV-3101PC spectrophotometer (Shimadzu Corporation, Kyoto, Japan). The element contents in the samples and solutions were analyzed using a Vista AX ICP-AES/MS (Varian Australia Pty Ltd., Mulgrave, Australia) inductively coupled plasma spectrometer. N_2_ adsorption-desorption isotherms were performed on a Micromeritics Tristar II 3020 (Micromeritics Instrument Corp., Norcross, GA, USA) surface area and porosity analyzer at 77 K. The DLS size and surface zeta potential of the nanoparticles in the suspensions were determined with a Zetasizer Nano-series (Malvern Instruments Ltd., Worcestershire, UK). Thermogravimetry (TG) curves were measured using a Netzsch STA 449C (Netzsch-Gerätebau GmbH, Selb, Germany) microanalyzer under flowing air with a heating rate of 10 K·min^−1^. The magnetization curve was measured at room temperature under a varying magnetic field using a Vibrating Sample Magnetometer (VSM) (LinkPhysics Corporation, Shanghai, China).

### 2.5. Leaching of Fe^3+^ from the L-Fe-NR

For checking the Fe^3+^ leaking from L-Fe-NR, dialysis of the L-Fe-NR aqueous solution (5 mg L-Fe-NR/20 mL of ultrapure water with a resistivity of 18.2 MΩ-cm) was carried out at 37 °C for a week using the dialysis tubes (MW: 3500, MD44). The Fe^3+^ content in the obtained filtrate was measured by ICP-MS analysis.

### 2.6. MRI Performance Test

The MR imaging properties in vitro were measured on a 3.0 T Siemens Magnetom Trio system (Siemens corporation, Erlangen, Germany) using a T_1_-weighted FSE-XL/90 sequence under the following parameters: TR/TE = 1000, 2000, 3000, 4000/7.9 ms; FOV = 18 cm^2^; matrix: 128 × 128; NEX = 2; slice thickness = 2 mm; space = 0.5 mm; coil: QUADKNEE. Aqueous suspensions of L-Fe-NR and L-Fe with different Fe^3+^ concentrations were placed into a series of 1.0 mL tubes for MR imaging tests. The iron contents of L-Fe-NR and L-Fe were determined by ICP-AES. The T_1_ and T_2_ values of the samples were recorded at different concentrations and plotted as 1/T_1_ or 1/T_2_ vs. the molar concentration of iron in L-Fe-NR and L-Fe suspensions, respectively. Then, the slopes of these lines provided the longitudinal relaxivity value r_1_ and the transverse relaxivity value r_2_, respectively.

### 2.7. Drug Loading and Release

A total of 40 mg of L-Fe-NR was dispersed in 120 mL of IBU alcohol/water (1:1 *v/v*) mixed solution (1 mg·mL^−1^). Then, the mixed solution was stirred for 24 h under dark conditions. The obtained L-Fe-NR-IBU was centrifuged, washed with alcohol/water (1:1 *v/v*) mixed solvent, and freeze-dried. The IBU loading capacity in the L-Fe-NR was obtained from the TG analysis results of L-Fe-NR-IBU and L-Fe-NR. Release kinetics of the loaded IBU from L-Fe-NR (40 mg) in PBS (400 mL, 0.2 M) with a pH value of 5.0, 6.0, and 7.4 at 37 °C were measured using the dialysis tubes (MW: 3500, MD44). The dialysis tubes were placed into a shaking table at 37 °C with a rotational speed of 150 rpm. A total of 3 mL of release medium was taken out at a given time interval with a syringe, analyzed by UV-Vis spectroscopy at a wavelength of 264 nm, and then replaced with the same volume of fresh release medium. The signal intensity values of extracted medium at 264 nm, which are directly proportional to the IBU concentrations in release medium, were gathered to calculate the IBU release percentages at different time intervals. The concentration of released IBU is calculated according to the following equation [43,44,45]:

$$C_{tcorr}=C_{t}+\frac{v}{V}\overset{t-1}{\underset{0}{\sum }}C_{t}$$

where *C_tcorr_* is the corrected IBU concentration in the release medium at time *t*, *C_t_* is the apparent concentration of the extracted release medium at time *t*, *v* is the volume of extracted release medium, and *V* is the total volume of release medium.

### 2.8. Cell Culture

Mouse macrophage line RAW 264.7 cells, human cervical cancer HeLa cells, and mouse L929 fibroblastic cells were cultured in Dulbecco’s modified Eagle’s medium (DMEM) containing 10% fetal bovine serum (FBS), 100 units·mL^−1^ penicillin, and 100 μg·mL^−1^ streptomycin at 37 °C in a humidified atmosphere of 5% CO_2_ and 95% air.

### 2.9. Cytotoxicity Assay of L-Fe-NR

The in vitro cytotoxicity of L-Fe-NR against RAW 264.7 cells, HeLa cells, and L929 cells was evaluated by MTT assays. RAW 264.7 cells, HeLa cells, and L929 cells were seeded in a 96-well microculture plate with 1 × 10^4^ cells and 0.1 mL of culture medium per well and cultured in 5% CO_2_ at 37 °C for 24 h, respectively. Then, L-Fe-NR suspensions with various concentrations (31.3, 62.5, 125, 250, 500, and 1000 μg·mL^−1^) were placed into the culture medium. After incubation for 24 h and 48 h, respectively, the culture media containing L-Fe-NR was decanted, and 100 μL of MTT solution (5 mg/mL) was added to each well and the plates were incubated for another 4 h. The culture medium was then replaced with 100 μL of DMSO per well, and the absorbance at 490 nm for each well was measured on a microplate reader (Bio-Tek ELx800 (BioTek Instruments Inc., Winooski, VT, USA)). The relative cell viabilities (%) at various concentrations were expressed as the percentage of untreated control cells ([A_absorbance_]_tested_/[A_absorbance_]_control_ × 100) [46]. The experiments were replicated three times and the cell viabilities were presented as the mean ± SD.

## 3. Results and Discussion

**Figure 1:**
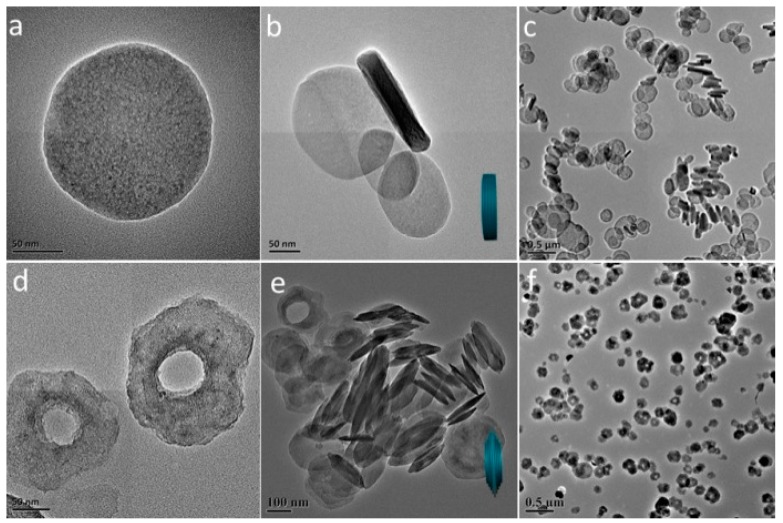
(**a**–**c**) TEM images of L-Fe; (**d**–**f**) L-Fe-NR at varied magnifications showing the morphology change after the etching.

**Figure 2:**
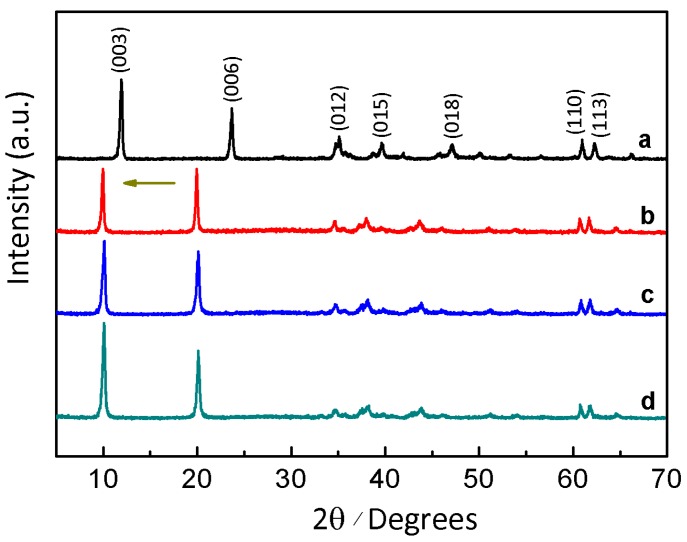
(**a**) XRD pattern of samples L-Fe; (**b**) L-Fe-AE-1; (**c**) L-Fe-AE-2; (**d**) L-Fe-NR showing the significant peak position shifts after the etching due to the substitution of NO_3_^−^ for CO_3_^2−^ in the interlayers.

**Figure 3:**
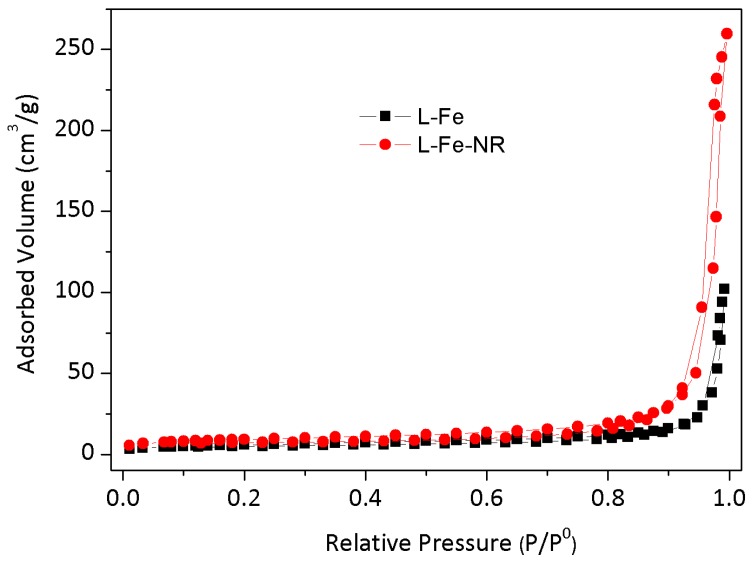
N_2_ adsorption-desorption isotherms of L-Fe and L-Fe-NR.

The L-Fe nanoparticles prepared by the coprecipitation method have a regular and relatively uniform discoid shape (Figure 1a–c). The XRD pattern (Figure 2a) shows a typical LDH structure of L-Fe (PDF card: #51-1525) and the calculated (001) basal spacing is 7.4 Å, demonstrating that the interlayer anion of L-Fe is CO_3_^2−^. The DLS analysis results show that L-Fe has an average DLS diameter of 266.1 nm with a low polydispersion index (PDI) of 0.078 (Figure S1). The surface areas calculated from N_2_ adsorption-desorption isotherms (Figure 3) and the zeta potential of L-Fe nanoparticles are 17.4 m^2^·g^−1^ and 41.0 mV (Figure S2), respectively. The L-Fe-NR was obtained through the acid etching of L-Fe in HNO_3_/NaNO_3_ solution of certain concentrations. Figure 1d–f show the TEM images of obtained L-Fe-NRs with a smaller size, thinner edge, and better dispersion than L-Fe. The L-Fe-NR retains the LDH structure (Standard PDF card: #51-1525) and all the diffraction peak positions shift toward lower angles compared with L-Fe (Figure 2d), and the calculated (001) basal spacing is 8.7 Å, implying that NO_3_^−^ in the acidic solution has replaced CO_3_^2−^ and been incorporated in the interlayers [47]. Compared with L-Fe, the XRD peak intensity of L-Fe-NR does not become weakened, demonstrating that L-Fe-NR retains a well-crystallized layered structure after acid etching, in spite of a large amount of dissolution and morphology change of the initial L-Fe in the acid etching process. The L-Fe-NR has a smaller DLS average size of 94.5 nm than L-Fe (Figure S1), which is due to the reduced particle size by etching and the better dispersion of L-Fe-NR in water. The zeta potential of L-Fe-NR is 37.8 mV and its surface area has been enhanced to 32.9 m^2^·g^−1^ (Figure S2 and Table 1). The Mg, Al, and Fe molar proportions in L-Fe and L-Fe-NR were measured by ICP-AES and are given in Table 2. From L-Fe to L-Fe-NR, the contents of Fe and Al increase from 32.7% and 1.3% to 33.8% and 2.8%, respectively, and consequently that of Mg decreases from 66.0% to 63.4%. The Mg/(Al + Fe) molar ratio dropped from 1.94 to 1.73, demonstrating that Mg^2^^+^ in the L-Fe is more easily dissolved compared with Al^3^^+^ and Fe^3^^+^ in the acid-etching process, which is due to the higher solubility product constant (K_sp_) of Mg(OH)_2_ than that of Al(OH)_3_ and Fe(OH)_3_, respectively.

**Figure 4:**
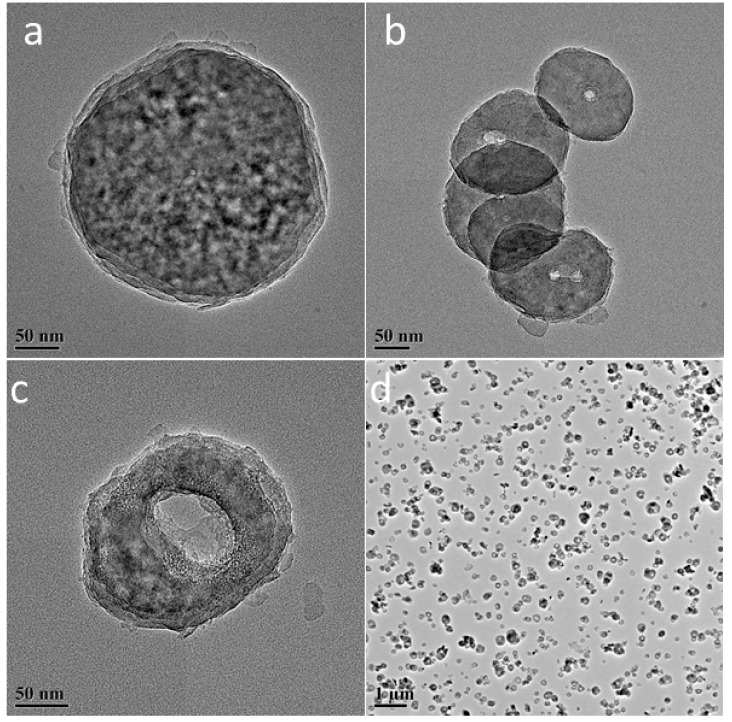
(**a**) TEM images of L-Fe-AE-1; (**b**) L-Fe-AE-2; (**c**,**d**) L-Fe-NR.

**Figure 5:**
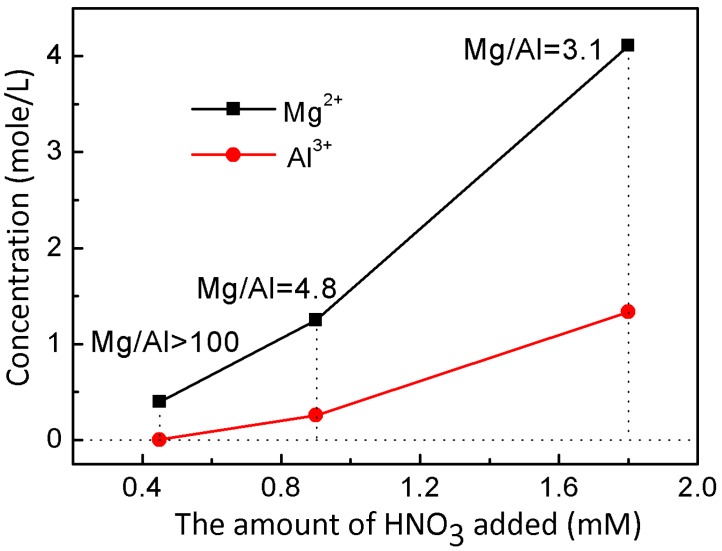
The Mg and Al concentrations in acidic etching solutions with varied amounts of HNO_3_ added.

To examine the properties of L-Fe during the acid etching process, various amounts of HNO_3_ solutions (65~68%) were added to the L-Fe suspension (100 mg L-Fe in 100 mL 0.75 M NaNO_3_ solution), respectively. After the acid etching, the obtained samples are denoted as L-Fe-AE-1, L-Fe-AE-2, and L-Fe-NR, with increased amounts of HNO_3_ added. The TEM images of L-Fe-AE-1 and L-Fe-AE-2 show that the initial dissolutions of L-Fe took place at the edge and/or centre (Figure 4a,b). The TEM images of L-Fe-NR obtained by using the highest concentration of HNO_3_ show a more obvious dissolution of L-Fe at the centre and edge zones, as compared to those of L-Fe-AE-1 and L-Fe-AE-2, and the substantially enhanced water dispersibility (Figure 4c,d), which is mainly due to the diminished aggregation effect via the preferential surface/interface dissolution of/among L-Fe nanoparticles. The strong diffraction peaks in XRD patterns of L-Fe-AE-1, L-Fe-AE-2, and L-Fe-NR demonstrate that L-Fe retained a well-crystallized layered structure after the acid etching (Figure 2b–d). The zeta potentials of the three samples are almost the same (Figure S2 and Table 1), which is in accordance with their stable layered structure phase. Meanwhile, the DLS average size decreases and the surface area increases gradually with increased amounts of HNO_3_ in etching solutions (Table 1, Figures S1 and S3), which is mainly because the edge and centre of L-Fe nanoparticles and the interface among L-Fe nanoparticles in agglomerates were dissolved in the acid etching process and thereby the particle size decreased and more new surface formed. The amount of dissolved Mg and Al from L-Fe in acidic etching solutions is presented in Figure 5, where the molar ratio of Mg to Al (Mg/Al) in the acidic etching solution changes from >100 to 3.1 with an increased amount of HNO_3_ and all of the results exceed the stoichiometric molar ratio in L-Fe (Mg/Al ≈ 2), indicating that more Mg^2+^ ions are dissolved than those of Fe^3+^ and Al^3+^ in the acidic etching solution, resulting in the relatively increased Fe^3+^ and Al^3+^ contents in the L-Fe-NR, as shown in Table 2.

**Figure 6:**
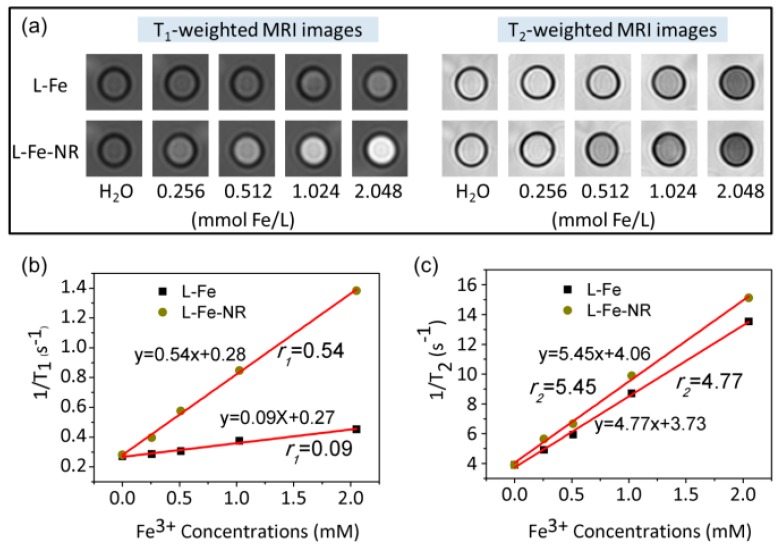
(**a**) The T_1_-weighted MRI images of aqueous suspensions of L-Fe and L-Fe-NR at different concentrations; (**b**) T_1_; (**c**) T_2_ relaxivity plots of aqueous suspensions of L-Fe and L-Fe-NR vs. Fe concentration.

The in vitro MR imaging properties of L-Fe-NR and L-Fe were measured on a 3.0 T Siemens Magnetom Trio system. T_1_-weighted and T_2_-weighted MR imagings of the L-Fe-NR and L-Fe suspensions with various Fe^3+^ concentrations and the control sample water are shown in Figure 6a. A positive T_1_-weighted MRI signal enhancement and a negative T_2_-weighted MRI signal enhancement can be observed for all the L-Fe and L-Fe-NR samples in comparison to the control sample, and the MRI signals were enhanced for the increased Fe^3+^ concentrations. Figure 6b,c show the longitudinal and transverse relaxation rates (1/T_1_ and 1/T_2_) dependence on Fe^3+^ concentration. The measured r_1_ value of the L-Fe-NR is 0.54 mM^−1^·s^−1^, much higher than that of L-Fe (0.09 mM^−1^·s^−1^). This could be ascribed to the higher surface area of L-Fe-NR (32.9 m^2^/g) than L-Fe (17.4 m^2^/g), and the relatively concentrated Fe^3+^ ions on the surface of L-NR-Fe compared to L-Fe as described above became accessible to water molecules, which induces the shortening of the longitudinal relaxation time T_1_ and the enhancement of the r_1_ value and T_1_-weighted MR imaging effect. The r_2_ values of L-Fe-NR and L-Fe are 5.44 mM^−1^·s^−1^ and 4.77 mM^−1^·s^−1^, respectively, and the very low r_2_ values are due to the low magnetic moment of the Fe^3+^ ions doped in the lattice (Figure S4) and weak magnetic inhomogeneity around the particles. The r_2_/r_1_ ratios of L-Fe-NR and L-Fe are 10.1 and 53.0, respectively. Such a low r_2_/r_1_ value of the L-Fe-NR indicates that L-Fe-NR could be applied as an efficient T_1_-weighted MRI contrast agent.

**Figure 7:**
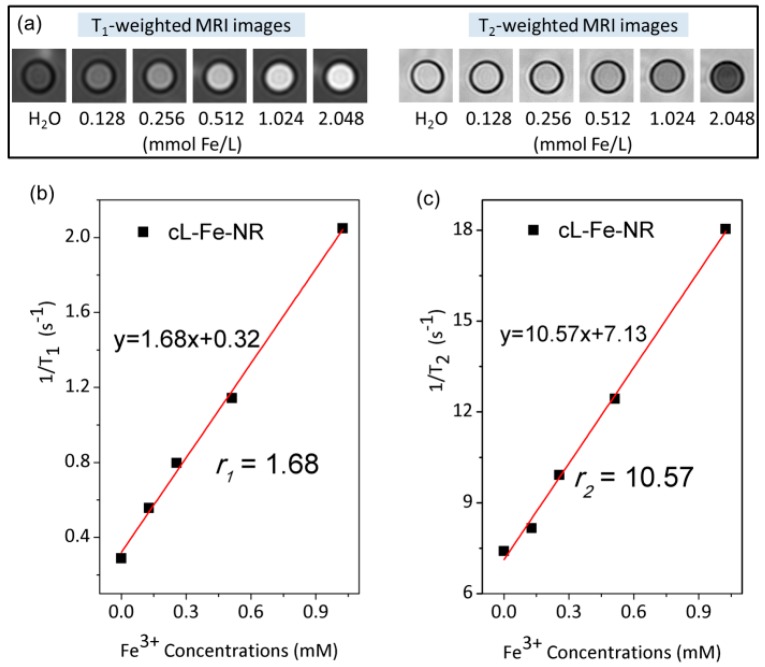
(**a**) The T_1_-weighted MRI images of aqueous suspensions of calcined samples of cL-Fe-NR at different concentrations; (**b**) T_1_; (**c**) T_2_ relaxivity plots of aqueous suspensions of cL-Fe-NR vs. Fe concentration.

Although the relaxivity values of L-Fe-NR are lower than those of the commercial Gd^3+^-based MRI contrast agents and Gd^3+^ doped LDH, they are comparable to the values obtained from part of Fe^3+^-doped materials and MnO nanoparticles (Table 3). In consideration of the nontoxic nature of the L-Fe-NR, the lower sensitivity may be compensated for by Fe^3+^ concentration enhancement. We then obtained a calcined sample of cL-Fe-NR by heating L-Fe-NR at 500 °C for 2 h, which is a MgO-Al_2_O_3_-Fe_2_O_3_ mixed oxide decomposed from mixed metal hydroxides, and found that cL-Fe-NR has a dramatically enhanced r_1_ value of 1.68 (Figure 7), as compared to that of L-Fe-NR, and a lowered r_2_/r_1_ ratio of 6.3, whilst maintaining high dispersibility (Figure S5). The enhanced T_1_-weighted MRI performance can probably be attributed to the fact that the Fe^3+^ in the less compact structure of cL-Fe-NR, derived from the decomposition of metal hydroxide components of L-Fe-NR, is more accessible to water molecules, which results in the shortening of the longitudinal relaxation time T_1_ and enhancement of the r_1_ value and T_1_-weighted MR imaging effect.

**Figure 8:**
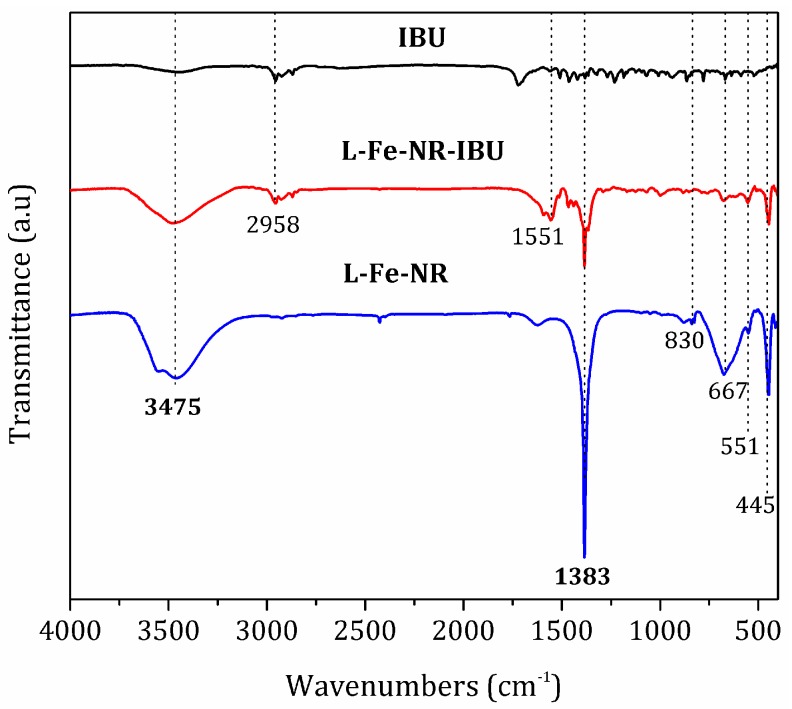
FT-IR spectra of IBU, L-Fe-NR, and L-Fe-NR-IBU.

**Figure 9:**
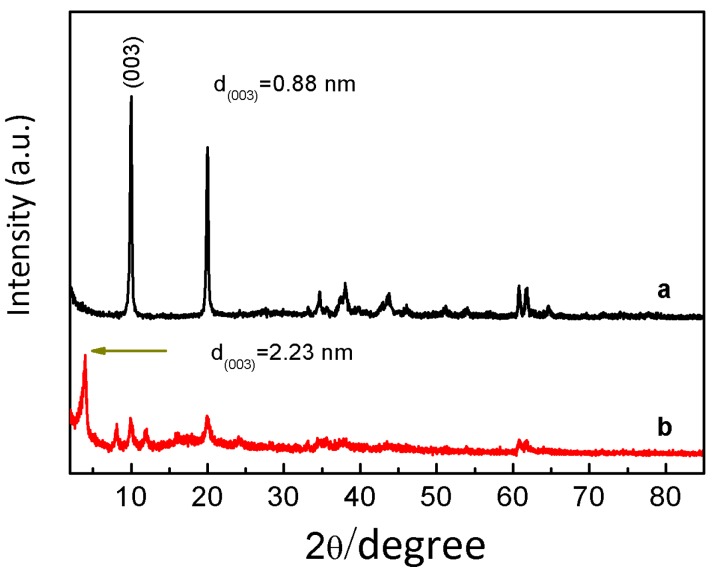
(**a**) XRD patterns of L-Fe-NR; (**b**) L-Fe-NR-IBU.

**Figure 10:**
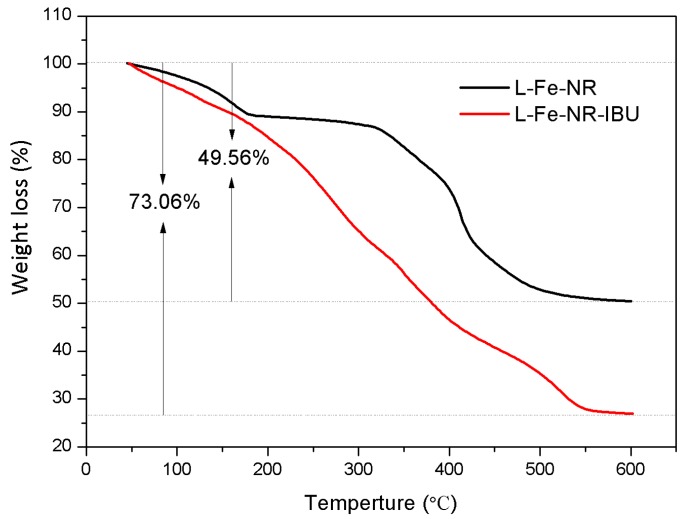
TG curves of L-Fe-NR and L-Fe-NR-IBU.

**Figure 11:**
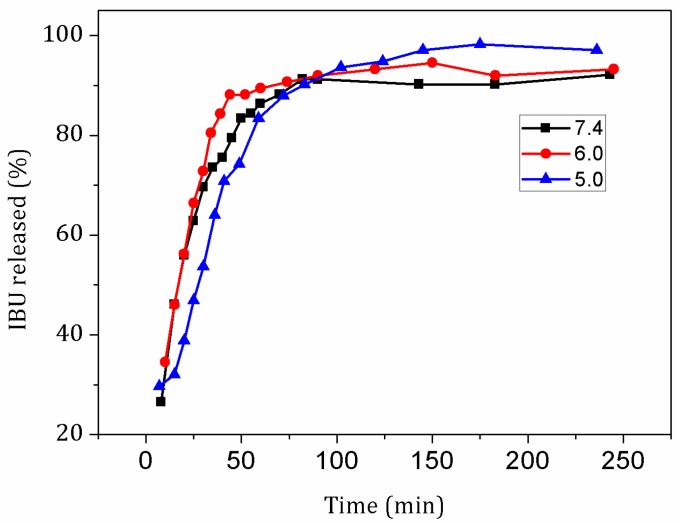
In vitro release profiles of L-Fe-NR-IBU under different pH values.

To testify the drug loading and release properties of L-Fe-NR, a common anti-inflammatory drug, ibuprofen (IBU), was chosen as a model drug. Figure 8 shows the FT-IR spectra of IBU, L-Fe-NR, and L-Fe-NR-IBU. The broad band centered around 3475 cm^−1^ is associated with the O-H stretching vibrations of the hydroxyl groups in L-Fe-NR. The bands at ca. 1383 cm^−1^ and 830 cm^−1^ are due to the ν_3_ and ν_2_ modes of interlayer nitrate ions in L-Fe-NR. The shoulder peak at 551 cm^−1^ and the sharp peak at 445 cm^−1^ correspond to metal-oxygen lattice vibrations in L-Fe-NR. The broad band at 667 cm^−1^ can be attributed to M-O-H bending in L-Fe-NR. The bands at ca. 2958 cm^−1^ and 1551 cm^−1^ are assigned to characteristic vibrations of the alkyl chain and the carboxylate group of intercalated ibuprofen anions, respectively [51,52]. L-Fe-NR-IBU has all the typical bands of both L-Fe-NR and IBU except the band of interlayer nitrate ions, demonstrating that the interlayer nitrate ions have been replaced by IBU in the L-Fe-NR-IBU. The XRD pattern of L-Fe-NR before and after drug loading is shown in Figure 9. After the loading of IBU, the diffraction peak positions of L-Fe-NR-IBU shift toward lower angles compared to L-Fe-NR, and the (001) basal spacing changes from 8.8 Å to 22.3 Å, further implying that anionic IBU has been incorporated in the interlayers of L-Fe-NR [53]. Figure 10 is the TG curves of L-Fe-NR and L-Fe-NR-IBU. The mass losses of L-Fe-NR and L-Fe-NR-IBU are 49.56% and 73.06%, respectively, and the calculated IBU loading capacity is 872.6 mg IBU/1000 mg L-Fe-NR. The in vitro drug release test was carried out in phosphate buffer solution (PBS 0.2 M) with pH values of 5.0, 6.0, and 7.4, respectively (Figure 11). It can be seen that the IBU releasing speed at a pH of 5.0 was slightly lower than that at a pH of 7.4 and 6.0 at the initial stage, probably due to the low pH decreasing the solubility of IBU [54]. However, the releasing proportion increased at equilibrium with the decreased pH of release medium attributed to the structure dissolution of the LDH carrier in the low pH. In addition, the release rates in all the mediums were rapid within the first 40 min, then gradually slowed down, and thereafter, equilibrium was reached. The rapid release of IBU from L-Fe-NR is presumed to be due to the anionic exchange between IBU and a great number of anions in the PBS (0.2 M), such as PO_4_^3−^_,_ Cl^−^, and especially CO_3_^2−^ derived from the dissolution of CO_2_ from air. Therefore, the L-Fe-NR can be used as an effective drug carrier with a high anionic drug loading capacity and sustained drug release profile.

**Figure 12:**
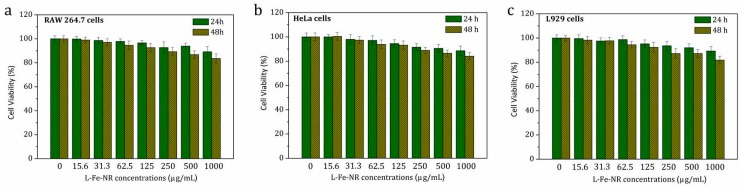
The viabilities: (**a**) RAW 264.7 cells; (**b**) HeLa cells; (**c**) L929 cells for 24 and 48 h respectively, when exposed to L-Fe-NR at different concentrations.

To determine the leakage of iron ions from L-Fe-NR, the filtrate was monitored by ICP-MS analysis after the dialysis of L-Fe-NR aqueous solution (5 mg/20 mL) for a week. The detected iron concentration in the filtrate is 2 ng/mL, demonstrating negligible iron ion leaching of L-Fe-NR. An in vitro MTT assay was carried out to investigate the cytotoxicity of the carriers. RAW 264.7 cells, HeLa cells, and L929 cells were incubated with L-Fe-NR of various concentrations for 24 and 48 h, respectively. Similar trends are found in the viabilities of these three cells (Figure 12). The cell viabilities gradually decreased with increasing L-Fe-NR concentrations and incubation times. However, even when co-cultured at a very high concentration of L-Fe-NR (1000 μg/mL), the viabilities of RAW 264.7 cells, HeLa cells, and L929 cells remained above 80% in 24 h and even 48 h of incubation, demonstrating the negligible cytotoxicity of L-Fe-NR. In addition, there are little differences between cell viabilities after 24 h and 48 h at low concentrations of L-Fe-NR, while the cell viability after 48 h obviously decreased in comparison to that after 24 h at high concentrations. As expected, a little change is observed in the viabilities of different cell lines, which may be attributed to the different physiological nature of the cells.

**Figure 13:**
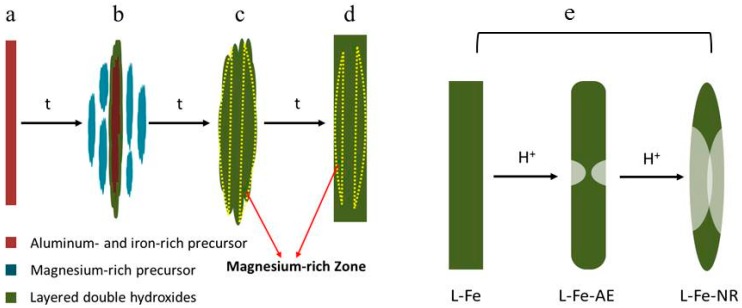
(**a**–**d**) Schematic illustration of the formation of L-Fe and (**e**) L-Fe-NR.

The nanoring formation in the acid etchant can be related to the non-uniform dissolutions of different parts of the discoid L-Fe, which is believed to originate from the compositional inhomogeneity in the microstructure of L-Fe. Such a compositional inhomogeneity formed during the coprecipitation process of L-Fe. During the preparation of L-Fe, alkali liquor was added dropwise into the Mg, Al, and Fe mixed nitrate solution until the solution pH value of 9.5, and the precipitate was then hydrothermally treated at 150 °C for 24 h. Both Al^3+^ and Fe^3+^ would precipitate with OH^−^ in the form of hydroxides more easily than Mg^2+^ because of the much lower solubility product constants of Al(OH)_3_ and Fe(OH)_3_ than that of Mg(OH)_2_. As the solution pH value increases, the Al- and Fe-rich hydroxide precursor co-precipitates first as a laminar structure (Figure 13a and Figure S7a,b), and the Mg-rich hydroxide precursor later forms and deposits on the surface of the Al- and Fe-rich precursor (Figure 13b and Figure S7c,d). Next during the hydrothermal treatment, both precursors crystallize into an LDH structure (Figure 13c and Figure S7e,f), accompanied by the homogenization of the element distribution as judged from the pure single phase of L-Fe in the XRD pattern (Figure 2a).

However, compositional inhomogeneity remains due to the tunable composition of the LDH structure to a certain extent and the incomplete homogenization of the elements between the precipitates successively formed, leading to the existence of an Mg-rich region in the centre of L-Fe disks, as schematically illustrated in Figure 13d. The preferential corrosion in the central Mg-rich region to Al- and Fe-rich regions, as well as at the edge part during acidic etching, leads to the final nanoring morphology of L-Fe-NR, as illustrated in Figure 13e.

## 4. Conclusions

In summary, Fe-doped Mg-Al-LDH nanorings have been successfully synthesized by a facile coprecipitation-acid etching route. The obtained nanoring has a small DLS average size of 94.5 nm, high dispersity, well-crystallized layered structure, high model drug loading capacity of 872.6 mg/g, and favourable T_1_-weighted MR imaging performance with an r_1_ value of 0.54 and low r_2_/r_1_ value close to 10, whilst maintaining low Fe^3+^ leakage and negligible cytotoxicity. The r_1_ value of calcined Fe-doped LDH nanoring can be further enhanced to as high as 1.68 with a considerably decreased r_2_/r_1_ value of 6.3. These results demonstrate the potential of Fe^3+^ doped LDH nanoring as both a T_1_-weighted MRI contrast agent and a simultaneous drug carrier. A possible mechanism has been proposed for the LDH nanoring formation, which may provide a reference for the synthesis of LDH with different morphologies. Finally, it should be noted that much work is still needed to realize the in vivo imaging of LDH-Fe, such as further modification to enhance the r_1_ value and increase the Fe content in LDH-Fe to ensure an adequate MRI signal intensity in vivo.

## Figures and Tables

**Table 1:** The DLS average sizes, zeta potentials, and BET surface areas (S_BET_) of L-Fe, L-Fe-AE-1, L-Fe-AE-2, and L-Fe-NR.

Sample	L-Fe	L-Fe-AE-1	L-Fe-AE-2	L-Fe-NR
DLS size (nm)	266.1	169.4	152.0	94.5
Zeta potential (mV)	41.0	40.1	40.1	37.8
S_BET_ (m^2^/g)	17.4	16.8	27.7	32.9

**Table 2:** The molar proportions of Mg, Al, and Fe and the molar ratios of Mg to (Al + Fe) in L-Fe and L-Fe-NR.

Sample	Mg	Al	Fe	Al + Fe	Mg/(Al + Fe)
L-Fe	66.0	32.7	1.3	34.0	1.94
L-Fe-NR	63.4	33.8	2.8	36.6	1.73

**Table 3:** The MRI relaxivity value comparisons of L-Fe-NR and cL-Fe-NR with Gd^3+^ doped LDH, other Fe^3+^ doped materials, and MnO nanoparticles.

Materials	r_1_ (mM^−^^1^·s^−^^1^)	r_2_ (mM^−^^1^·s^−^^1^)	r_2_/r_1_	Magnetic Field Strength	Imaging Type	Reference
L-Fe-NR	0.54	4.77	8.83	3.0 T	T1	This work
cL-Fe-NR	1.68	10.57	6.29	3.0 T	T1	This work
LDH-Gd(III)	2.4	-	-	3.0 T	T1	[26]
LDH-Gd(III)/Au	6.6	-	-	3.0 T	T1	[26]
CdTeS-Fe(III)	-	3.60	-	4.7 T	T2	[48]
C_3_N_4_-Fe(III)	3.10	24.40	7.87	3.0 T	T1	[49]
Fe@O-MWCNTs	0.20	25.00	125	7.1 T	T2	[50]
Fe(III)/Fe@O-MWCNTs	0.21	35.00	167	7.1 T	T2	[50]
MnO	0.81	-	-	3.0 T	T1	[38]
MnO@SiO_2_	1.34	-	-	3.0 T	T1	[38]
MnO	0.37	1.74	2.49	3.0 T	T1	[32]

## Supplementary Materials

The following are available online at www.mdpi.com/1996-1944/10/10/1140/s1, Figure S1: Dynamic light scattering (DLS) size distributions of L-Fe, L-Fe-AE-1, L-Fe-AE-2, and L-Fe-NR, Figure S2: Zeta potential distributions of L-Fe, L-Fe-AE-1, L-Fe-AE-2, and L-Fe-NR, Figure S3: N_2_ adsorption−desorption isotherms of L-Fe-AE-1, L-Fe-AE-2, and L-Fe-NR, Figure S4: Room temperature magnetization curves of L-Fe and L-Fe-NR, Figure S5: TEM images of cL-Fe-NR showing the preserved ring morphology and high dispersity, Figure S6: In vitro release profiles of L-Fe-NR-IBU in PBS solution at a pH value of 7.4, Figure S7: TEM images of L-Fe-Pre-1 (a,b), L-Fe-Pre-2 (c,d), and L-Fe-Pre-3 (e,f). L-Fe-Pre-1, L-Fe-Pre-2, and L-Fe-Pre-3 are the intermediates in the preparation of LDH, as described in the experimental section.
