# Supplementary material for: Synthesis and In Vitro Characterization of Fe3+-Doped Layered Double Hydroxide Nanorings as a Potential Imageable Drug Delivery System
Source: Materials (Basel). 2017 Sep 27;10(10):1140. doi: 10.3390/ma10101140 (PMC5666946; doi:10.3390/ma10101140)
Supplement: Supplementary file 1 [file materials-10-01140-s001.pdf]

Supporting information

Synthesis and in Vitro characterization of Fe^{3+}-doped layered double hydroxide nanorings as a potential imageable drug delivery system

Lijun Wang *, Yusen Wang, Xiaoxia Wang

School of Chemistry and Chemical Engineering, Shaoxing University, Shaoxing 312000, PR China

*Corresponding author. E-mail address: ljwang@usx.edu.cn

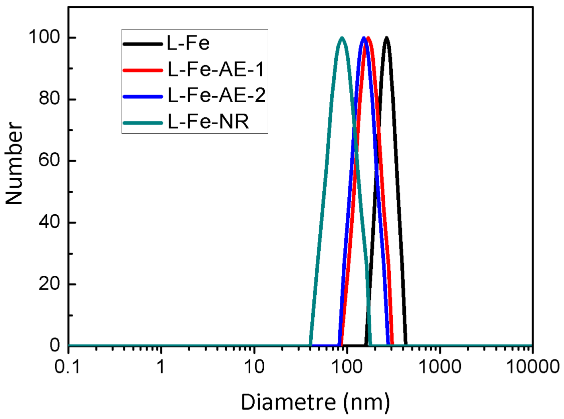

Figure S1. Dynamic light scattering (DLS) size distributions of L-Fe, L-Fe-AE-1, L-Fe-AE-2, and L-Fe-NR.

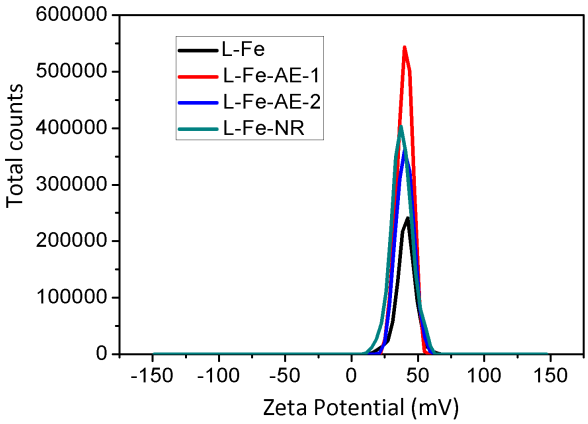

Figure S2. Zeta potential distributions of L-Fe, L-Fe-AE-1, L-Fe-AE-2, and L-Fe-NR.

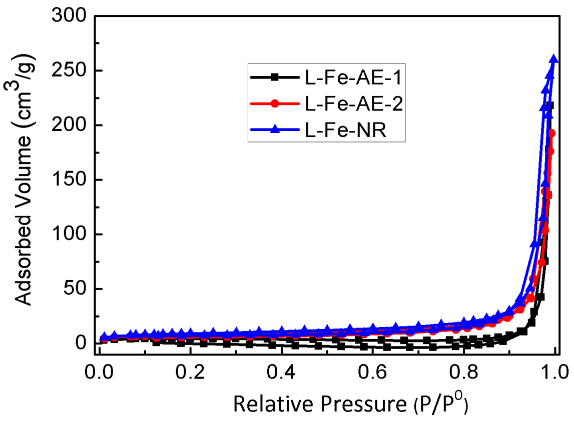

Figure S3. N2 adsorption−desorption isotherms of L-Fe-AE-1, L-Fe-AE-2, and L-Fe-NR.

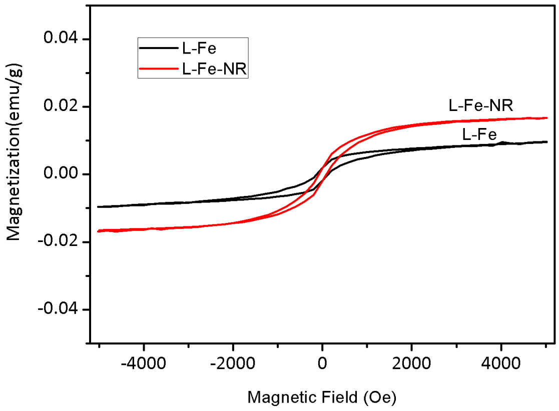

Figure S4. Room temperature magnetization curves of L-Fe and L-Fe-NR.

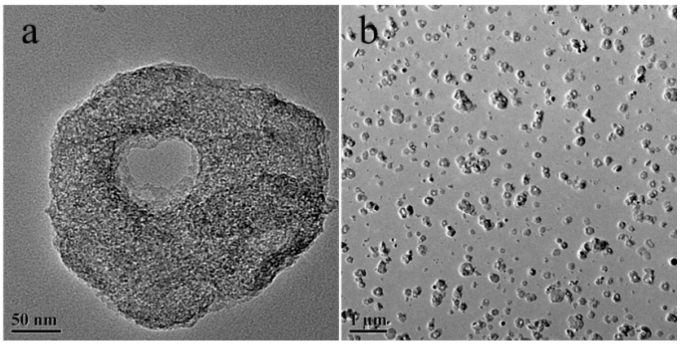

Figure S5. TEM images of cL-Fe-NR showing the preserved ring morphology and high dispersity.

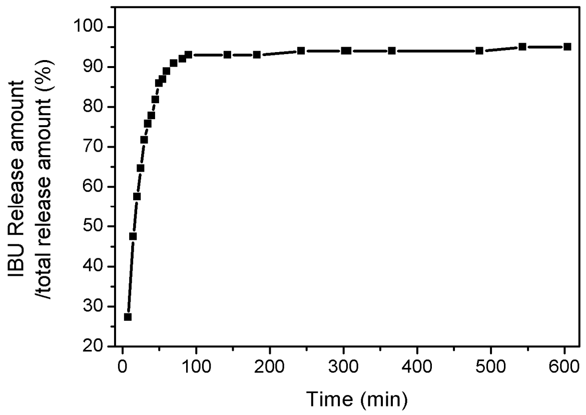

Figure S6. In vitro release profiles of L-Fe-NR-IBU in PBS solution at a pH value of 7.4.

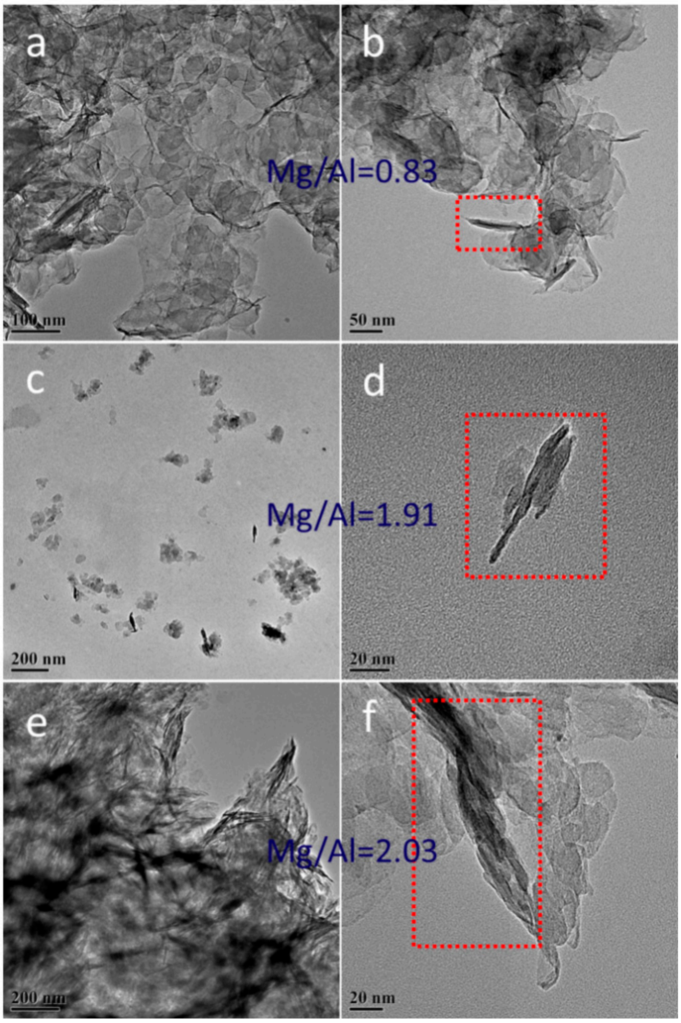

Figure S7. TEM images of L-Fe-Pre-1 (a, b), L-Fe-Pre-2 (c, d), and L-Fe-Pre-3 (e, f). L-Fe-Pre-1, L-Fe-Pre-2, and L-Fe-Pre-3 are the intermediates in the preparation of LDH, as described in the experimental section.
